# 5-FU and ixabepilone modify the microRNA expression profiles in MDA-MB-453 triple-negative breast cancer cells

**DOI:** 10.3892/ol.2013.1697

**Published:** 2013-11-22

**Authors:** YONGSHAN YAO, SHENGHAN CHEN, XIN ZHOU, LI XIE, AIJUN CHEN

**Affiliations:** 1 Third Department of General Surgery, The First College of Clinical Medical Science, China Three Gorges University, Yichang, Hubei 443003, P.R. China; 2 Department of Electrocardiography, The First College of Clinical Medical Science, China Three Gorges University, Yichang, Hubei 443003, P.R. China

**Keywords:** 5-FU, ixabepilone, luminal androgen receptor-type, triple-negative breast cancer, microRNA

## Abstract

This study aimed to discover new potential mechanisms of chemotherapy with drugs used in the treatment of luminal androgen receptor (LAR)-type triple-negative breast cancer (TNBC). We examined the microRNA (miRNA) expression profiles of LAR-type TNBC *in vitro*, and explored the variation in miRNA expression profiles in cells when treated with the chemotherapy drugs capecitabine and ixabepilone. The present study revealed that the expression levels of the three antitumor miRNAs, miR-122a, miR-145 and miR-205, were significantly elevated in MDA-MB-453 LAR-type TNBC tumor cells treated with 5-fluorouracil together with ixabepilone. By contrast, carcinogenic miR-296 miRNA expression significantly declined, and levels of several other miRNAs such as miR-221, miR-210, miR-21 and miR-10b were also altered. The drugs may exert their effects through the regulation of miRNA expression levels, thereby providing a theoretical basis for clinical implementation of miRNA expression profiles as a diagnostic method for the early diagnosis, classification and prognosis of breast cancer.

## Introduction

Triple-negative breast cancer (TNBC) is recognized as a subtype of breast cancer in which three receptors [estrogen receptor (ER), progesterone receptor and human epidermal growth factor receptor 2] are negatively expressed on the surface of breast cancer cells (1,2). TNBC is an invasive tumor with a high degree of malignancy, and accounts for approximately 12–20% of breast cancer cases (2–5). Compared with other breast cancer subtypes, the overall prognosis for TNBC is poor, as it is prone to brain metastasis and bone metastasis. Currently, according to genomic differences, TNBC is divided into three categories with a total of six subtypes: i) basal-like (BL), including BL1, BL2 and immunomodulatory (IM) subtypes; ii) mesenchymal-like (ML), including mesenchymal (M) and mesenchymal stem-like (MSL) subtypes; and iii) luminal androgen receptor (LAR) subtype (4,6–8). The genes involved in steroid synthesis and androgen metabolism are highly expressed in the LAR subtype of TNBC.

There are no uniform treatment guidelines and no standard chemotherapy program for TNBC to date (4,9–11). Chemotherapy is the only option for TNBC treatment and has a good prognosis for those TNBC patients who are sensitive to chemotherapy. Results from clinical observations have shown that the pathological complete remission rate of TNBC patients is higher than that in ER^+^ breast cancer patients after standard chemotherapy (treatment with anthracycline and taxane drugs) (4,9,12–16). However, therapeutic drugs are limited for chemotherapy-resistant TNBC patients, as they are resistant to common chemotherapy drugs for breast cancer, such as anthracycline and taxane (4,9,10). Capecitabine and ixabepilone have been used for the treatment of anthracycline and taxane chemotherapy-resistant TNBC patients. Preclinical studies have shown that capecitabine with ixabepilone has synergistic antitumor activity (17). As monotherapy with ixabepilone has a good human tolerance and synergistic antitumor activity without overlapping toxicities with capecitabine, ixabepilone monotherapy or combination therapy with capecitabine has become an effective treatment strategy for TNBC patients (4,9,10,18–25).

MicroRNAs (miRNAs) are a class of endogenous non-coding small RNAs that can inhibit gene expression at the post-transcriptional level by inhibiting mRNA translation and promoting mRNA degradation, and are involved in many key processes of cell activity, such as development, differentiation, metabolism, apoptosis and proliferation (26). An increasing number of studies have revealed that miRNA is involved in tumorigenesis, differentiation and metastasis, and that anticancer drugs can change the miRNA expression profiles of tumor cells, indicating that the change in miRNA expression profiles may account for the mechanism behind the antitumor effect of these chemotherapy drugs (27). Moreover, it has been reported that the chemotherapy drug-tolerance of tumors is also associated with miRNA expression (28). Therefore, miRNA has been used in studies on early tumor diagnosis, classification, prognosis, drug sensitivity prediction and exploration of drug mechanisms (26,28–36). To date, the abnormal expression of miRNA profiles has been found to exist in a variety of breast cancer types. It has been reported that miR-21, miR-155, miR-210, miR-29c, miR-196a, miR-213, miR-191, miR-203, miR-29b and miR-93 are highly expressed in breast cancer, while miR-125b, miR-145, miR-100, miR-10b, miR-125-b2, miR-497 and miR143 are minimally expressed, producing an anti-apoptotic effect, and promoting proliferation, metastasis and invasion by modulating the target gene expression (13,26,29–33). In Radojicic *et al*’s study on miRNA expression in TNBC, a significantly higher expression of miR-21, miR-210 and miR-221 and a notably lower expression of miR-10b, miR-145, miR-205 and miR-122a were observed compared with that in normal tissues. However, there were no statistically significant differences in miR-222 and miR-296 expression between TNBC and normal tissues (31). miRNA expression profiles have been recognized as a potential diagnostic method for tumors. However, the literature documenting the miRNA expression profiles of pathological specimens of TNBC subtypes has been limited to date.

The TNBC subtype cell line MDA-MB-231 is the most commonly used cell line in previous studies. However, these studies have rarely focused on miRNA expression profiles (34–36). There are very few reported studies on miRNA expression profiles for other TNBC subtypes (31). Capecitabine and ixabepilone can be used for the treatment of metastatic breast cancer (including TNBC), but no studies have stated the impact of these two drugs on miRNA expression profiles in TNBC. miRNA plays an essential role in early tumor diagnosis, classification, prognosis, anti-drug sensitivity forecast, drug action mechanism and cancer treatment; therefore, it is important to explore the miRNA profile expression of different TNBC subtypes and the changes in miRNA profile expression when various TNBC subtype cells are treated with chemotherapy drugs.

This study aimed to explore the miRNA profile expression differences between the LAR-type TNBC MDA-MB-453 cell line and normal breast cells, including six carcinogenic miRNAs (miR-296, miR-222, miR-221, miR-210, miR-10b and miR-21) and three antitumor miRNAs (miR-145, miR-205 and miR-122a). The effect of the capecitabine active metabolite 5-fluorouracil (5-FU), ixabepilone and 5-FU + ixabepilone on the miRNA profile expression of MDA-MB-453 was also explored, aiming to further clarify the association between miRNAs and LAR-type TNBC. In addition, we aimed to discover potential new mechanisms of chemotherapy with these drugs in LAR-type TNBC treatment.

## Materials and methods

### Cell culture

The human LAR-type TNBC MDA-MB-453 cell line and the normal breast CRL-2713 (MDA-kb2) cell line were obtained from ATCC Company (Manassas, VA, USA). MDA-MB-453 cells were cultured in RPMI-1640 (SH30809.01B; Hyclone Laboratories, Inc., Logan, UT, USA) and incubated at 37ºC with humidified air containing 5% CO_2_, while MDA-kb2 cells were cultured in Leibovitz’s L-15 medium (SH30525.01; Hyclone Laboratories, Inc.) with atmospheric air at 37ºC. Both types of medium were supplemented with 10% FBS (SV30087.02; Hyclone Laboratories, Inc.), penicillin (100 U/ml) and streptomycin (0.1 mg/ml).

### Drug treatment and MTT assay

MDA-MB-453 cells cultured in 96-well plates (3599; Corning, Tewksbury, MA, USA) were treated with either a gradient concentration of 5-FU (F6627; Sigma-Aldrich, St. Louis, MO, USA) ranging from 0.0001–10,000 μM or ixabepilone (Hubei Honch Pharmaceutical Co., Ltd., Wuhan, China) ranging from 0.0001–10,000 nM. The IC_10_ value of these drugs, the concentration that can induce 10% cell inhibition, was analyzed by an MTT assay and determined for further analysis. For the MTT assay, MDA-MB-453 cells were seeded in quadruplicate on 96-well plates at a density of 3×10^5^ cells per well in 100 μl RPMI-1640 medium, and incubated in an air-humidified incubator at 37ºC with 5% CO_2_ for 24 h. The cells were then treated with a gradient concentration of drugs and cultured for another 24 h. Subsequently, 20 μl of 5 mg/ml MTT (AR1156; Wuhan Boster Biological Technology, Wuhan, China) dissolved in PBS (BD-1070; Hubei Biossci, Wuhan, China) were added to each well and the cells were incubated for 4 h, followed by the addition of 100 μl of formazan and incubation for 30 min on a flat shaker at room temperature in the dark to completely dissolve the crystals. The optical density was determined by a microplate reader (RT-6100; Rayto Life and Analytical Sciences Co., Ltd, Shenzhen, China) using a 560-nM filter. The results were statistically analyzed and the cell inhibition rate was determined with the following formula: cell inhibition % = 100% − (OD_2_-OD_0_)/(OD_1_-OD_0_) × 100%. OD_0_, culture medium alone; OD_1_, cells untreated; OD_2_, cells treated with drugs.

### RNA extraction and stem-loop RT-PCR

Total RNA was extracted from MDA-MB-453 or MDA-kb2 cell lines using TRIzol reagent (15596026; Invitrogen Life Technologies, Carlsbad, CA, USA) according to the manufacturer’s instructions. Stem-loop RT-PCR assay was performed using an All-in-One™ First Strand cDNA Synthesis kit (AORT-100; GeneCopoeia, Inc., Rockville, MD, USA) as described previously (37). A total of 13 μl of reaction system was prepared containing extracted RNA (10 ng-1 μg), U6 primer and miRNA stem-loop primer, incubated at 65ºC for 10 min, and then incubated on ice for 2 min. The 13 μl of RNA-primer mixture was then mixed with 5 μl of 5X reaction buffer, 1 μl of dNTP (25 mM), 1 μl of Rnase inhibitor (15 U/μl) and 1 μl of M-MLV RTase (200 U/μl), and incubated at 42ºC for 60 min prior to heat-inactivation at 85ºC for 5 min. The product was stored at −20ºC for subsequent usage. The sequences of miRNA stem-loop primers are shown in Table I.

### qPCR

qPCR was conducted with a real-time PCR system (ABI Step One Plus; Applied Biosystems, Inc., Foster City, CA, USA) in 20 μl of reaction mixture containing 5 μl of cDNA, 10 μl of 2X All-in-One qPCR mix (AORT-1200; GeneCopoeia, Inc.), 0.4 μl of each forward and reverse primer and 50X ROX reference dye buffer. The PCR conditions were 3 min at 95ºC, and 40 cycles consisting of 15 sec at 95ºC, 20 sec at 56ºC and 20 sec at 72ºC. The internal control primer was designed with Primer 5.0 software (PREMIER Biosoft, Palo Alto, CA, USA) and the sequence of the common reverse primer for qPCR was 5′-GTGCAGGGTCCGAGGT-3′, while the sequences of the forward primers for the detection of miRNAs are shown in Table II.

### Statistical analysis

The data from the MTT assay were statistically analyzed by SPSS 11.5 software (SPSS, Inc., Chicago, IL, USA), and the data from the miRNA expression level detection were statistically analyzed by GraphPad Prism 5 software (GraphPad, San Diego, CA, USA). Comparisons between the two groups were performed by Student’s t test, while comparisons among multiple groups were performed by one-way analysis of variance. P<0.05 was considered to indicate a statistically significant difference.

## Results

### Effect of 5-FU and ixabepilone on the human LAR-type TNBC cell line, MDA-MB-453

**Figure 1:**
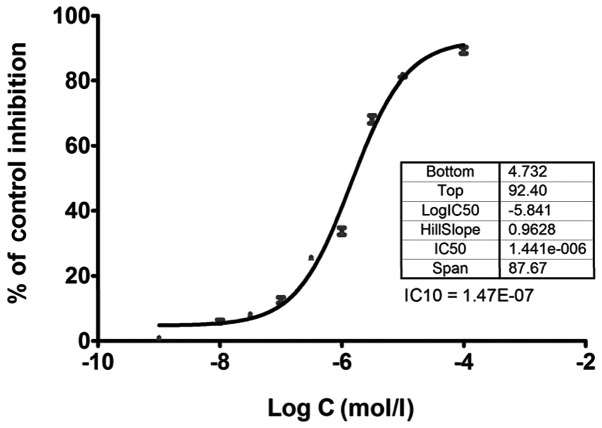
Dose-response curve of cell inhibition of 5-FU on the human LAR-type TNBC cell line, MDA-MB-453. IC_10_ of 5-FU was 1.47E-07 mol/l. 5-FU, 5-fluorouracil; LAR, luminal androgen receptor; TNBC, triple-negative breast cancer.

**Figure 2:**
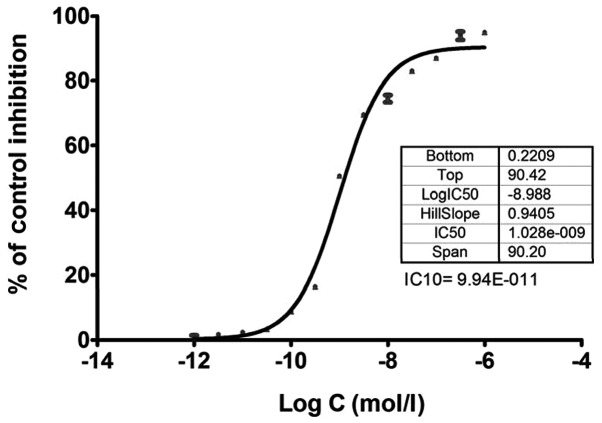
Dose-response curve of cell inhibition of ixabepilone on the human LAR-type TNBC cell line MDA-MB-453. IC_10_ of ixabepilone was 9.94E-011 mol/l LAR, luminal androgen receptor; TNBC, triple-negative breast cancer.

As 5-FU and ixabepilone have been implicated in the treatment of TNBC, the sensitivity of the LAR-type TNBC cell line, MDA-MB-453, to 5-FU and ixabepilone was determined and a dose-response curve was generated by detecting the cell viability with an MTT assay as shown in Figs. 1 and 2. The low concentration of 10% cell inhibition (IC_10_ value) by 5-FU and ixabepilone drugs was selected to further examine the effects of these two drugs on miRNA expression in MDA-MB-453 cells, as a high concentration of drugs may lead to cellular changes rather than genetic changes (27). The IC_10_ value of 5-FU was 1.47E-07 mol/l (Fig. 1), while that of ixabepilone was 9.94E-011 mol/l (Fig. 2).

### Alternation of three anti-tumor miRNA expression levels in MDA-MB-453 cells after 5-FU and ixabepilone treatment

**Figure 3:**
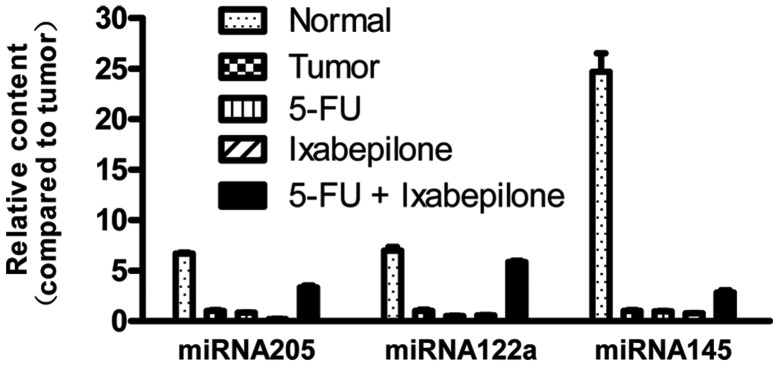
Expression features of three antitumor microRNAs in normal, tumor, 5-FU, ixabepilone and 5-FU + ixabepilone groups. 5-FU, 5-fluorouracil.

A previous study demonstrated that expression levels of miR-122a, miR-145 and miR-205 in TNBC are significantly lower than those in normal tissues, and that these three miRNAs have antitumor effects (31). In this study, we analyzed the expression levels of miR-122a, miR-145 and miR-205 in normal breast MDA-kb2 cells and MDA-MB-453 cells prior to and after 5-FU and ixabepilone treatment. As shown in Fig. 3, the expression levels of miR-122a, miR-145 and miR-205 in MDA-MB-453 cells were significantly lower than those in normal breast cells (P<0.05, Table III). When MDA-MB-453 cells were treated with 5-FU or ixabepilone, the expression levels of these three miRNAs marginally decreased (Fig. 3, Table III). However, when tumor cells were treated with 5-FU together with ixabepilone, the expression levels increased significantly (P<0.05, Fig. 3, Table III).

### Alternation of six carcinogenic miRNA expression levels in MDA-MB-453 cells after 5-FU and ixabepilone treatment

**Figure 4:**
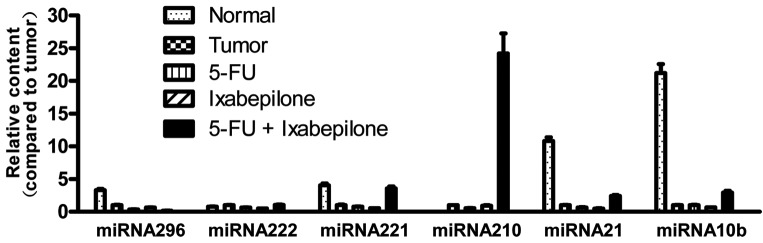
Expression features of six carcinogenic microRNAs in normal, tumor, 5-FU, ixabepilone and 5-FU + ixabepilone groups. 5-FU, 5-fluorouracil.

It has been reported that miR-296, miR-222, miR-221, miR-210, miR-10b and miR-21 are abnormally expressed in breast cancer, and are recognized as carcinogenic miRNAs. In the present study, we examined expression levels of these six miRNAs in normal breast MDA-kb2 cells and MDA-MB-453 cells. In Fig. 4, we can observe that the expression of miR-210 in MDA-MB-453 cells was at an undetected level while that of miR-222 was marginally higher than in normal cells. Expression levels of miR-296, miR-221, miR-21 and miR-10b in MDA-MB-453 cells were notably lower than those in normal cells (P<0.05, Fig. 4, Table IV). When MDA-MB-453 cells were treated with 5-FU or ixabepilone, expression levels of all six miRNAs were marginally decreased compared with those in untreated tumor cells (Fig. 4, Table IV). However, when cells were treated with 5-FU + ixabepilone, expression levels of miR-221, miR-210, miR-21 and miR-10b increased significantly, while expression levels of miR-296 showed a significant decrease (P<0.05, Fig. 4, Table IV). No significant difference was observed between expression levels of miR-222 after treatment with 5-FU + ixabepilone compared with those in the untreated tumor cells (P>0.05, Table IV).

## Discussion

Breast cancer is the most common type of malignant tumor in women worldwide. Approximately 1.2 million women suffer from breast cancer each year, of whom around 500,000 die, and the incidence is growing at a rate of 2% per annum (38). As a systemic disease, the therapeutic effect of breast cancer depends on early diagnosis, the degree of metastasis and treatment options (3,4,12,13). Generally, the early stage of breast cancer is estrogen receptor (ERα)-positive and non-metastatic, so an early diagnosis is vital for effective treatment of breast cancer. However, breast cancer patients in the earlier stages usually lack clear clinical characteristics and there are currently no clinically effective auxiliary diagnostic methods for early breast cancer detection (3,29). Therefore, the majority of cases are diagnosed as advanced tumors with ineffective treatment options and a poor prognosis (16). Although certain diagnostic tools and biomarkers are currently being used for the clinical diagnosis of breast cancer, there are many shortcomings: Ionizing radiation and a high false-positive rate exist in breast X-ray examination techniques, and tumor markers such as ERα lack specificity (3). Therefore, detecting the early signs of breast cancer and developing early diagnostic reagents are essential for the primary prevention of breast cancer.

Increasing evidence has revealed that miRNA expression profiles can be used to identify the tissue samples that are difficult to be determined by histology, since miRNA expression profiles may represent the degree of differentiation of tissues (3). Therefore, the establishment of miRNA expression profiles in normal tissue and tumor tissue is crucial for the accurate and efficient diagnosis of the disease. The first detailed study of the correlation between miRNA and breast cancer was reported by Croce’s group (39). In their study, they detected 76 types of miRNA expression in breast cancer by miRNA microarray analysis. The results showed that, compared with normal tissue, the expression levels of 29 miRNAs significantly changed in breast cancer. They found that five miRNAs are required for the 100% successful identification of normal tissue and cancerous tissue: miR10b, miR-125b, miR-145, miR-21 and miR-155. Moreover, the expression levels of miR-125b and miR-145 decreased markedly while the other three miRNAs showed a clear increase (39). Currently, there is increasing evidence to suggest that microRNA analysis may be implemented in breast cancer diagnosis, prognosis and treatment (40–42).

However, few studies have revealed the miRNA expression profiles of pathological specimens of TNBC subtypes (31). Studies on TNBC have often not carefully distinguished the TNBC subtypes, resulting in a certain degree of confusion when studying TNBC. In the present study, we examined miRNA expression in the LAR-type TNBC cell line, MDA-MB-453, and explored the variations in this expression compared with the normal breast cell line, MDA-kb2. Six carcinogenic miRNAs (miR-296, miR-222, miR-221, miR-210, miR-10b and miR-21) and three antitumor miRNAs (miR-145, miR-205 and miR-122a) were analyzed. The results showed that the expression levels of miR-122a, miR-145 and miR-205 in MDA-MB-453 cells were significantly lower than those in normal breast cells, as has been observed in other subtypes of TNBC (31). However, the expression levels of miR-296, miR-221, miR-21 and miR-10b in MDA-MB-453 cells were also notably lower than those in normal cells, which is in contrast to previous studies on miRNA expression profiles in other subtypes of TNBC (31). This indicates that different TNBC subtypes exhibit different microRNA expression features, which can be used for early diagnosis and classification.

Capecitabine is an oral nucleoside metabolic inhibitor that can be metabolized to 5-FU in tissue to block DNA synthesis (43). Preclinical studies have shown that capecitabine combined with ixabepilone has synergistic antitumor activity, and that the effect of this combination therapy on metastatic TNBC is superior to a single drug application (4,9,10,18–25). However, the mechanisms underlying this effect remain unclear. We hypothesized that combination therapy with these two drugs may change miRNA expression profiles in tumor cells, thereby efficiently inhibiting tumor growth via modulation of target gene expression. It has been reported that antitumor drugs can change miRNA expression profiles in tumor cells, and that chemotherapy drug-resistant tumors are also correlated with miRNA expression level. Therefore, the variation in miRNA expression profiles may be one mechanism by which drug treatment inhibits tumors (27,28). Studies by Shah *et al* have found that variations in miRNA expression profiles occur in 5-FU- treated breast cancer MCF-7 cells (27), prompting us to explore the miRNA expression profiles in LAR-type TNBC MDA-MB-453 cells that have been treated with capecitabine and ixabepilone, alone or in combination. In the present study, we examined the expression levels of nine miRNAs and found that when MDA-MB-453 cells were treated with 5-FU or ixabepilone alone, the expression of almost all miRNAs decreased marginally. By contrast, following combined treatment with 5-FU and ixabepilone, the expression of seven miRNAs increased significantly, the expression levels of miR-296 decreased and those of miR-222 showed no notable change. Although the expression levels of three antitumor miRNAs (miR-145, miR-205 and miR-122a) increased significantly when tumor cells were treated with 5-FU + ixabepilone, those of other carcinogenic miRNAs (miR-221, miR-210, miR-10b and miR-21) also increased. The treatment effect of 5-FU + ixabepilone on LAR-type TNBC therefore requires further study. The abovementioned results indicate that joint treatment with 5-FU and ixabepilone had a more marked effect on LAR-type TNBC MDA-MB-453 cells compared with treatment with 5-FU or ixabepilone alone. Moreover, it significantly altered the miRNA expression profiles in tumor cells, indicating that the examination of miRNA expression profiles could be used for prognosis.

TNBC has a variety of subtypes. In this study, we examined the miRNA expression profiles of LAR-type TNBC MDA-MB-453 cells, and analyzed the correlation between miRNA expression profiles in tumor cells and in normal breast MDA-kb2 cells. We found that the expression levels of nine microRNAs in LAR-type TNBC were unlike those previously reported in other subtypes of TNBC. Therefore, it is necessary to distinguish the subtypes of TNBC during treatment. We also explored the variation in miRNA expression profiles in TNBC cells when treated with the chemotherapy drugs capecitabine and ixabepilone, alone or in combination, in order to clarify the potential mechanisms of chemotherapy in LAR-type TNBC. Our study provides a theoretical basis for the future clinical application of miRNA expression profiles in the early diagnosis, classification and prognosis of breast cancer.

## Figures and Tables

**Table I:** Sequences of microRNA stem-loop primers.

microRNA	microRNA sequence	microRNA stem-loop primer
296	AGGGCCCCCCCTCAATCCTGT	GTCGTATCCAGTGCAGGGTCCGAGGTATTCGCACTGGATACGACacagga
222	AGCTACATCTGGCTACTGGGT	GTCGTATCCAGTGCAGGGTCCGAGGTATTCGCACTGGATACGACacccag
221	AGCTACATTGTCTGCTGGGTTTC	GTCGTATCCAGTGCAGGGTCCGAGGTATTCGCACTGGATACGACgaaacc
210	CTGTGCGTGTGACAGCGGCTGA	GTCGTATCCAGTGCAGGGTCCGAGGTATTCGCACTGGATACGACtcagcc
10b	TACCCTGTAGAACCGAATTTGTG	GTCGTATCCAGTGCAGGGTCCGAGGTATTCGCACTGGATACGACcacaaa
21	TAGCTTATCAGACTGATGTTGA	GTCGTATCCAGTGCAGGGTCCGAGGTATTCGCACTGGATACGACtcaaca
145	GTCCAGTTTTCCCAGGAATCCCT	GTCGTATCCAGTGCAGGGTCCGAGGTATTCGCACTGGATACGACagggat
205	TCCTTCATTCCACCGGAGTCTG	GTCGTATCCAGTGCAGGGTCCGAGGTATTCGCACTGGATACGACcagact
122a	TGGAGTGTGACAATGGTGTTTG	GTCGTATCCAGTGCAGGGTCCGAGGTATTCGCACTGGATACGACcaaaca

**Table II:** Sequences of forward primers for the detection of microRNAs.

microRNA	Forward primers of microRNA
296	AGGGCCCCCCCTCAA
222	CTGGGTGTCGTATCCAGTGC
221	TTGTCTGCTGGGTTTCGTCG
210	GTGTGACAGCGGCTGAGT
10b	AGAACCGAATTTGTGGTCGT
21	TCAGACTGATGTTGAGTCGT
145	TTCCCAGGAATCCCTGTCGT
205	TCCACCGGAGTCTGGTCGTAT
122a	TGTGACAATGGTGTTTGGTCG

**Table III:** P-values of comparisons of three antitumor microRNA expression levels between the tumor group and normal or drug-treated groups.

	P-value			
	
microRNA	Normal vs. tumor	5-FU vs. tumor	Ixabepilone vs. tumor	5-FU+ ixabepilone vs. tumor
miRNA122a	<0.0001	0.0171	0.0305	<0.0001
miRNA145	0.0002	0.7914	0.0853	0.0020
miRNA205	<0.0001	0.2423	0.0022	0.0004

**Table IV:** P-values of comparisons of six carcinogenic microRNA expression levels between the tumor group and normal or drug-treated groups.

	P-value			
	
microRNA	Normal vs. tumor	5-FU vs. tumor	Ixabepilone vs. tumor	5-FU+ ixabepilone vs. tumor
miRNA10b	<0.0001	0.9165	0.0039	0.0022
miRNA21	<0.0001	0.0097	0.0009	0.0015
miRNA210	-	<0.0001	0.4806	0.0016
miRNA221	0.0006	0.1021	0.0090	0.0013
miRNA222	0.0141	0.0027	0.0006	0.6478
miRNA296	0.0007	0.0016	0.0182	0.0005
